# Trained immunity in cancer and autoimmunity: a double-edged sword in immune memory reprogramming

**DOI:** 10.3389/fimmu.2026.1782830

**Published:** 2026-03-18

**Authors:** Nasrin Salari, Mehrshad Shams, Fatemeh Tavassoli Razavi, Esmaeil Yazdanpanah, Valentyn Oksenych, Dariush Haghmorad

**Affiliations:** 1 Cancer Research Center, Semnan University of Medical Sciences, Semnan, Iran; 2 Department of Immunology, School of Medicine, Semnan University of Medical Sciences, Semnan, Iran; 3 Department of Immunology, School of Medicine, Mazandaran University of Medical Sciences, Sari, Iran; 4 Immunology Research Center, Mashhad University of Medical Sciences, Mashhad, Iran; 5 Oslo Bioconsulting, Oslo, Norway; 6 Department of Microbiology, Danylo Halytsky Lviv National Medical University, Lviv, Ukraine

**Keywords:** autoimmune diseases, cancer immunotherapy, epigenetic reprogramming, innate immune memory, trained immunity

## Abstract

Trained immunity, characterized by the long-term functional reprogramming of innate immune cells through epigenetic and metabolic modifications, has emerged as a pivotal concept bridging innate and adaptive immune responses. This review explores the dual role of trained immunity as both a protective mechanism in cancer and a pathogenic driver in autoimmune diseases. We first discuss the underlying mechanisms involving histone modifications, chromatin remodeling, and metabolic pathways such as glycolysis and the mTOR/HIF-1α axis, alongside key regulators including NOD2 and pattern recognition receptors. The contribution of trained immunity to antitumor responses is highlighted through its ability to enhance innate cell cytotoxicity, remodel the tumor microenvironment, and synergize with immune checkpoint blockade and BCG immunotherapy. Conversely, we examine how infections, dysbiosis, and dietary factors can induce maladaptive trained immunity, leading to persistent hyperinflammatory states and exacerbation of autoimmune diseases such as rheumatoid arthritis, systemic lupus erythematosus, and multiple sclerosis. Furthermore, we address therapeutic strategies to modulate trained immunity, including small molecules, β-glucan, statins, and BCG derivatives, emphasizing their potential applications in cancer immunotherapy and autoimmunity control. We also underscore the risks of unintended immune activation, such as autoimmune flare-ups during cancer treatment or compromised host defense during immunosuppression. Finally, we discuss future directions, including the development of trained immunity-based vaccines, personalized immunomodulatory approaches, and the integration of multi-omics and artificial intelligence to design patient-specific interventions. Understanding the complex interplay between trained immunity, cancer, and autoimmunity will be crucial for translating these insights into innovative therapeutic strategies.

## 1 Introduction

Innate immunity provides an immediate response to invading pathogens, whereas adaptive immunity generates a slower but highly targeted response through antigen receptor gene rearrangement that establishes immunological memory. This classical distinction has become less clear with the discovery of pattern recognition receptors (PRRs), which enable innate immune cells to exhibit a degree of specificity in microbial recognition (1). Adaptive immunity achieves immunological memory through experience-driven antibody diversity, in contrast to the rapid defense mediated by innate effectors such as phagocytes and cytokines. However, this dichotomy has been challenged by the ability of PRRs to allow innate immune sentinels to discriminate between foreign and self-ligands, conferring a limited but important level of selective pathogen detection similar to aspects of adaptive immunity (2). Although extensive research has examined innate immune function, recent investigations reveal that this first line of defense is far more complex than previously understood (3, 4). Evidence of protection from reinfection has been observed not only in flora and invertebrates lacking adaptive immunity but also in mammals (5, 6). These findings have led to the concept that innate immunity can be reprogrammed by prior exposure to pathogens or their components, a phenomenon known as trained immunity or innate immune memory.

Trained immunity differs from classical immunological memory in several ways. First, it involves specific cell types such as myeloid cells, natural killer (NK) cells, and innate lymphoid cells (ILCs) and relies on germline-encoded effector molecules and recognition pathways, including PRRs and cytokines, rather than antigen-specific lymphocytes (7). Second, while classical memory is based on clonal expansion and gene rearrangement, trained immunity enhances responsiveness to subsequent stimuli in a nonspecific manner. This heightened state is driven by transcriptional changes and epigenetic reprogramming, which modify gene expression without altering the DNA sequence (8). Finally, trained immunity induces a functionally altered state in innate immune cells that persists for weeks to months after the initial stimulus, compared with the years-long persistence of classical immunological memory (9).

Tuberculosis (TB), caused by Mycobacterium tuberculosis, remains a leading cause of global mortality. In 2017, TB affected 10 million individuals and caused 1.3 million deaths (10). To combat TB, Albert Calmette and Camille Guérin developed the bacillus Calmette–Guérin (BCG) vaccine in 1921, which has since been incorporated into most national vaccination programs. BCG was derived from an attenuated strain of Mycobacterium bovis that underwent serial passages and accumulated over 14 genomic deletions (11). Beyond TB prevention, BCG vaccination has been associated with reduced childhood mortality due to nonspecific protection against unrelated infections (12, 13).

Studies have shown that BCG-vaccinated children are less likely to develop acute lower respiratory tract infections (ALRI) compared with unvaccinated children (14). Additionally, epidemiological data from West Africa indicate that BCG vaccination reduces mortality by over 40 percent, primarily through protection against diseases such as malaria, sepsis, respiratory infections, and leprosy (15–18). Repeated BCG vaccination has also been reported to lower HbA1c levels and eliminate insulin-autoreactive T cells in type 1 diabetes (19). Multiple sclerosis (MS) is a progressive autoimmune disease characterized by inflammatory demyelination of the central nervous system (CNS) (20). Although no curative treatment exists, a small clinical study involving 12 MS patients demonstrated that BCG vaccination might reduce CNS lesion activity (21).

Further support for trained immunity in vertebrates comes from experimental studies. Mice vaccinated with BCG developed T-cell-independent protection against secondary infections with Candida albicans and Schistosoma mansoni (22, 23). Similarly, epidemiological surveys have reported that live vaccines such as BCG, measles, and oral polio induce nonspecific protective responses against infections unrelated to their intended targets (24). Proof-of-concept trials in healthy adults vaccinated with BCG, along with clinical studies in BCG-immunized newborns and those co-administered with the hepatitis B vaccine, support these findings (25–27). Importantly, the nonspecific protective effects of BCG have been utilized in cancer therapy, including treatment of bladder cancer, melanoma, leukemia, and lymphoma (28–31).

Although acute inflammation plays a role, long-term innate immune memory induced by BCG appears to be a major contributing factor. Recent research links BCG’s anticancer efficacy to trained immunity and enhanced NK cell activity, as vaccinated individuals exhibit increased cytokine production upon heterologous stimulation (32, 33).

Trained immunity, characterized by long-term pro-inflammatory reprogramming of innate immune cells driven by epigenetic and metabolic changes, including increased aerobic glycolysis and cytokine production, represents a promising strategy to overcome the immunosuppressive tumor microenvironment (TME) and augment antitumor T-cell activity in cancer immunotherapy. This dual nature underscores the need for cautious therapeutic modulation of trained immunity. A critical concern is the overlap between the metabolic pathways underlying trained immunity and those exploited by cancer. The mTOR/HIF-1α-dependent aerobic glycolysis necessary for trained immunity induction is also a hallmark of tumor metabolism (34, 35). Similarly, the mevalonate pathway involved in trained immunity is hijacked by cancer cells to support their growth (36). This shared metabolic reliance suggests that therapies targeting these pathways could inadvertently promote tumor progression or worsen inflammatory autoimmune conditions. Moreover, cytokines central to trained immunity induction, such as IFN-γ, IL-1β, and TNF-α, are well-known mediators of autoimmune diseases including lupus and rheumatoid arthritis (RA). Repeated stimulation with trained immunity agonists like BCG or β-glucan may potentially enhance autoreactive immune responses and contribute to the progression of such conditions.

Trained immunity, characterized by long-term pro-inflammatory reprogramming of innate immune cells driven by epigenetic and metabolic changes, represents a context-dependent phenomenon whose outcome is determined by stimulus type, tissue microenvironment, and hematopoietic programming level (37). In cancer, microbial stimuli such as BCG or β-glucan typically induce antitumor myelopoiesis characterized by enhanced glycolysis and IL-1β production, whereas tumor-derived metabolites such as lactate, prostaglandin E2, and hypoxia-driven HIF-1α activation may skew myeloid differentiation toward immunosuppressive phenotypes including myeloid-derived suppressor cells (MDSCs) (38). This mechanistic duality underscores the importance of defining contextual determinants when therapeutically targeting trained immunity.

## 2 Mechanisms of trained immunity

### 2.1 Epigenetic changes: histone modifications and chromatin accessibility

Epigenetic changes regulate gene expression without altering the DNA sequence, forming an epigenetic code that determines which genes are activated and when. These mechanisms are essential for development, homeostasis, and disease regulation (39). Epigenetic modifications include DNA methylation, histone post-translational modifications (HPTMs), regulation by non-coding RNAs (ncRNAs), and RNA modifications (40, 41).

Histones, positively charged nuclear proteins, are structural components of chromatin. Core histones (H2A, H2B, H3, H4) form an octamer around which DNA wraps to create nucleosomes, while linker histone H1 stabilizes nucleosomes and compacts chromatin (42). HPTMs, including acetylation, methylation, crotonylation, lactylation, and succinylation, influence chromatin structure and gene transcription. Acetylation and crotonylation generally promote chromatin relaxation and gene activation, whereas specific methylation or sumoylation marks repress transcription (43).

Chromatin accessibility reflects how open DNA is for transcription factor binding. It is dynamically regulated by histone modifications and chromatin remodeling complexes. High-throughput techniques such as ATAC-seq and DNase-seq show that open chromatin regions are enriched at active promoters and enhancers, linking accessibility to transcriptional control (44). Importantly, such epigenetic mechanisms are critical for establishing trained immunity by sustaining gene expression programs that enhance innate immune cell responsiveness.

### 2.2 Metabolic rewiring: glycolysis and oxidative phosphorylation

Metabolic reprogramming enables cells to adjust their energy production in response to environmental changes. A prominent example is the Warburg effect, where tumor cells favor glycolysis over oxidative phosphorylation even in the presence of oxygen, leading to lactate accumulation (45). This metabolic shift supports rapid proliferation and also affects gene expression through histone lactylation and chromatin remodeling (46). Immune cells undergo similar metabolic adaptations. Activated T cells primarily depend on glycolysis to meet their energy needs, while memory and regulatory T cells rely more on oxidative phosphorylation and fatty acid oxidation. Within nutrient-deprived tumor microenvironments, competition for glucose and lactate buildup impairs cytotoxic T-cell and NK-cell activity, weakening antitumor immunity and facilitating immune evasion (46).

Metabolic reprogramming in trained immunity can be divided into induction and maintenance phases. During the induction phase, engagement of receptors such as Dectin-1 activates the Akt–mTOR–HIF-1α axis, leading to increased glycolysis, cholesterol biosynthesis via the mevalonate pathway, and accumulation of metabolites that support histone modifications such as H3K4me3 (47). Pharmacologic inhibition of glycolysis with 2-deoxyglucose or blockade of mTOR with rapamycin has been shown to prevent the establishment of trained phenotypes in monocytes, indicating that glycolytic flux is essential for induction (47).

In contrast, the maintenance phase is characterized by sustained chromatin accessibility at inflammatory loci and persistent metabolic bias in hematopoietic progenitors. Although glycolysis is critical for establishing trained immunity, long-term persistence appears to rely more heavily on stable epigenetic remodeling than on continuous metabolic activation. These distinctions are important when considering therapeutic interventions aimed at reversing pathogenic trained immunity (48).

### 2.3 Key regulators: NOD2, mTOR, and HIF-1α

Trained immunity involves sustained functional reprogramming of innate immune cells closely linked to metabolic changes. HIF-1α plays a central role in this process, particularly under hypoxic conditions typical of the tumor microenvironment. By upregulating genes such as GLUT1, LDHA, and PDK1, HIF-1α drives glycolysis and lactate production, features shared by trained myeloid responses and tumor-associated immune modulation (49). The mTOR pathway, especially mTORC1, senses environmental and nutrient signals upstream of HIF-1α. mTORC1 activation stabilizes HIF-1α and enhances glycolytic metabolism, supporting M1 macrophage polarization, glycolytic flux, and pro-inflammatory cytokine production—hallmarks of trained immunity observed in cancer and autoimmune contexts (50, 51). NOD2, a cytosolic pattern recognition receptor, mediates trained immunity through recognition of muramyl dipeptide (MDP) and induction of epigenetic modifications. However, its influence on metabolic rewiring is context-dependent. In monocytes exposed to Mycobacterium tuberculosis, NOD2 deficiency did not impair glycolytic activation, suggesting that NOD2 may contribute to trained immunity through mechanisms beyond direct metabolic regulation (52). Together, these regulatory pathways demonstrate that trained immunity is orchestrated through tightly interconnected metabolic and epigenetic circuits, reinforcing its context-dependent impact in cancer and autoimmune diseases.

## 3 Trained immunity in cancer

The functional outcome of trained immunity in cancer is highly dependent on contextual determinants including stimulus type, metabolic environment, and tissue niche. Microbial-derived training stimuli such as BCG or β-glucan often promote M1-like polarization, enhanced antigen presentation, and cytotoxic activity (53). In contrast, tumor-derived metabolites including lactate and lipid intermediates may reprogram myelopoiesis toward immunosuppressive MDSCs (54). Thus, trained immunity should not be viewed as uniformly antitumor or protumor, but rather as a spectrum shaped by environmental cues.

Evidence supporting trained immunity in cancer derives from diverse experimental systems, including β-glucan–trained human monocyte *in vitro* models (55), BCG-vaccinated murine melanoma and lung cancer models, lupus-prone autoimmune mouse models with secondary tumor implantation, and hematopoietic stem cell reprogramming studies following intravenous BCG administration (56, 57). These models have identified sustained H3K4me3 enrichment at inflammatory gene promoters, increased glycolytic flux, and enhanced myelopoiesis as mechanistic hallmarks of trained immunity in tumor settings (58). By specifying cellular subsets and model systems, these findings provide mechanistic insight into how innate memory influences tumor immunity.

In melanoma, trained immunity has demonstrated significant antitumor effects. For example, the β-glucan molecule ABB i16 induces monocyte training and suppresses melanoma growth in mouse models (55). Similarly, the CTB subunit metabolically reprograms dendritic cells, while MTP10-HDL improves the efficacy of immune checkpoint inhibitors (ICIs) by re-educating myeloid cells to control melanoma progression (55, 59).

In lung cancer, yeast-derived β-glucan particles (WGP) enhance innate immune activation. These particles induce durable activation of interstitial macrophages, increasing their phagocytic and cytotoxic activity and reducing both primary tumors and metastases (60).

In gastric cancer, Helicobacter pylori infection induces a unique form of trained immunity characterized by NF-κB-dependent monocyte hyper-responsiveness. Unlike typical microbial tolerance, this imprinting sustains chronic inflammation and promotes tumorigenesis (61, 62).

Despite its therapeutic benefits, trained immunity can also facilitate tumor progression under specific conditions. In liver cancer, hyperlipidemia and DNA hypomethylation promote metabolic reprogramming that triggers pro-inflammatory signaling, fibrosis, and hepatocellular carcinoma (63, 64). Similarly, oxidative stress, inflammasome activation, and IL-1 family cytokines contribute to a tumor-permissive microenvironment in chronic liver disease (65, 66). In lung cancer, macrophage-driven inflammation, induced by surfactant phosphatidylcholines or viral infections, establishes an immunosuppressive TME that supports tumor growth and immune evasion (67, 68). Elevated cytokines such as IL-6, commonly produced by trained innate immune cells, further promote cancer cell survival and invasiveness (69, 70).

In non-muscle invasive bladder cancer (NMIBC), intravesical BCG administration following transurethral resection significantly reduces tumor recurrence and progression (71, 72) BCG disrupts tumor-associated immune suppression by recruiting macrophages, CD4^+^ T helper cells, CD8^+^ T cells, and NK cells, leading to robust local inflammation (73–75). Furthermore, bladder tumor cells infected with BCG express antigen-presenting and co-stimulatory molecules, enabling them to serve as targets for cytotoxic immune responses (76). The antitumor effects of BCG are also linked to its ability to induce local inflammation and disrupt tumor-mediated immune tolerance (77). Intravenous BCG reprograms hematopoietic stem and progenitor cells (HSPCs), enhances myelopoiesis, and increases CD8^+^ T-cell expansion while reducing myeloid-derived suppressor cells (MDSCs) (78, 79). However, prolonged intravesical BCG therapy has also been associated with tumor-promoting immunosuppressive changes in the TME (80).

Innate immune memory is now recognized in multiple cell types, including monocytes, macrophages, NK cells, neutrophils, ILCs, and polymorphonuclear leukocytes. These cells, which lack antigen receptor gene rearrangement, rely on PRRs for detecting pathogen-associated molecular patterns (PAMPs) and damage-associated molecular patterns (DAMPs) (81).

Neutrophils trained with β-glucan display increased degranulation and reactive oxygen species (ROS) generation, directly contributing to tumor cell lysis (82). Type I interferon signaling mediates their antitumor effects, while inflammatory cytokines recruit additional immune effectors, amplifying the antitumor immune response. This response correlates with glycolytic reprogramming consistent with the Warburg effect (35).

Similarly, trained monocytes and macrophages undergo histone acetylation and methylation, adopt glycolytic metabolism, and exhibit elevated secretion of cytokines such as TNF-α, IL-1β, IL-6, and IL-12. This promotes immune cell recruitment and tumor cytotoxicity. In macrophages, activation of the sphingosine-1-phosphate (S1P) pathway and Drp1-mediated mitochondrial fission enhances phagocytic and cytotoxic activity, skewing macrophages toward an M1-like phenotype with robust antigen presentation and co-stimulatory molecule expression (CD80, CD86) (60). This conditioning is particularly effective against stromal-rich cancers like pancreatic cancer, where immune infiltration is limited (83, 84).

NK cells, crucial for targeting MHC class I-deficient tumor cells, exhibit enhanced cytotoxicity following cytokine conditioning (IL-12, IL-15, IL-18) or BCG vaccination. These trained NK cells produce increased IFN-γ, synergizing with CD8^+^ T cells to amplify antitumor immunity (33, 85).

**Figure 1:**
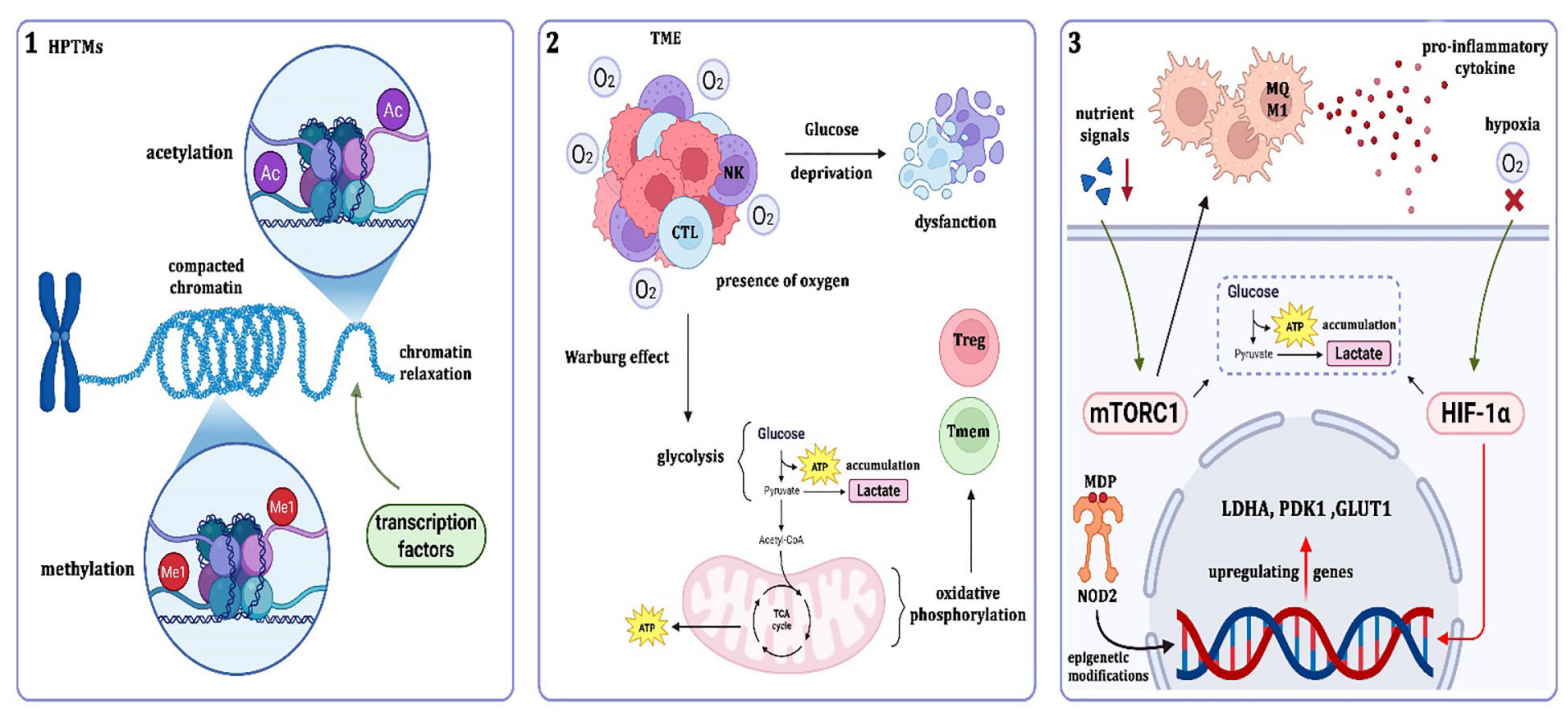
Epigenetic and metabolic mechanisms underlying trained immunity.

Conversely, MDSCs represent a major immunosuppressive barrier. They inhibit T-cell activity via ROS/NO generation (86, 87), amino acid depletion through arginase-1 and indoleamine 2,3-dioxygenase (IDO) (88, 89), immune checkpoint induction (PD-L1, VISTA) (90, 91), and regulatory T-cell recruitment via CCR5 (92). Pathogens such as HIV, Mycobacterium tuberculosis, and Trypanosoma cruzi exploit MDSC expansion to evade immunity (93). Vaccines including BCG and influenza mRNA vaccines may inadvertently promote MDSC accumulation, attenuating T-cell responses (94, 95) (**Figure 1**).

Collectively, these findings indicate that trained immunity in cancer operates along a functional spectrum, with its antitumor or protumor outcome determined by metabolic context and myeloid differentiation cues.

(1) Epigenetic reprogramming: Histone post-translational modifications (HPTMs) such as acetylation (Ac) and methylation (Me) regulate chromatin compaction and relaxation, thereby controlling transcription factor accessibility and gene expression. (2) Metabolic rewiring: In the tumor microenvironment (TME), immune cells experience glucose deprivation and oxygen fluctuations. Tumor and immune cells undergo the Warburg effect, favoring glycolysis and lactate accumulation over oxidative phosphorylation. This metabolic shift affects T cell subsets (CTL, Treg, Tmem) and natural killer (NK) cell function, ultimately shaping antitumor or immunosuppressive responses. (3) Key regulators: Nutrient and inflammatory signals activate the mTORC1–HIF-1α axis, upregulating glycolytic genes such as LDHA, PDK1, and GLUT1. Additionally, NOD2, through recognition of muramyl dipeptide (MDP), induces epigenetic modifications that sustain long-term immune reprogramming. Together, these interconnected pathways link epigenetic regulation, metabolic adaptation, and innate immune memory, with implications for both cancer progression and therapeutic intervention.

## 4 Trained immunity in autoimmune diseases

### 4.1 Triggers: infections, microbiota, diet

Trained immunity is an enhanced, non-specific immune response by innate immune cells following an initial stimulus. This response is mediated by metabolic and epigenetic changes in bone marrow progenitor or tissue-resident cells. In autoimmune and autoinflammatory diseases, trained immunity contributes to persistent inflammation and disease exacerbation. Factors such as infections, gut microbiota, and diet act as key modulators of innate immune training and influence disease progression (96).

Infections represent a major trigger of trained immunity in autoimmune disorders. The innate immune system detects PAMPs through PRRs such as TLRs and NOD-like receptors (NLRs), rapidly inducing inflammation. However, chronic infections can sustain this activation, leading to aberrant B and T cell responses against self-antigens. Neutrophil extracellular traps (NETs) further amplify inflammation by releasing DNA and self-antigens. Moreover, viruses such as EBV and HCMV can induce cytokines (e.g., IFN-α, TNF-α) or mimic self-antigens (molecular mimicry), thereby activating autoreactive immune cells and breaking immune tolerance (97).

Dysbiosis of the gut microbiota also plays a pivotal role. The loss of beneficial SCFA-producing species, such as Faecalibacterium prausnitzii, weakens mucosal tolerance and drives low-grade systemic inflammation. Dysbiosis further affects hematopoiesis by reprogramming myeloid progenitors toward a pro-inflammatory phenotype, thereby exacerbating autoimmune disease activity (98).

**Figure 2:**
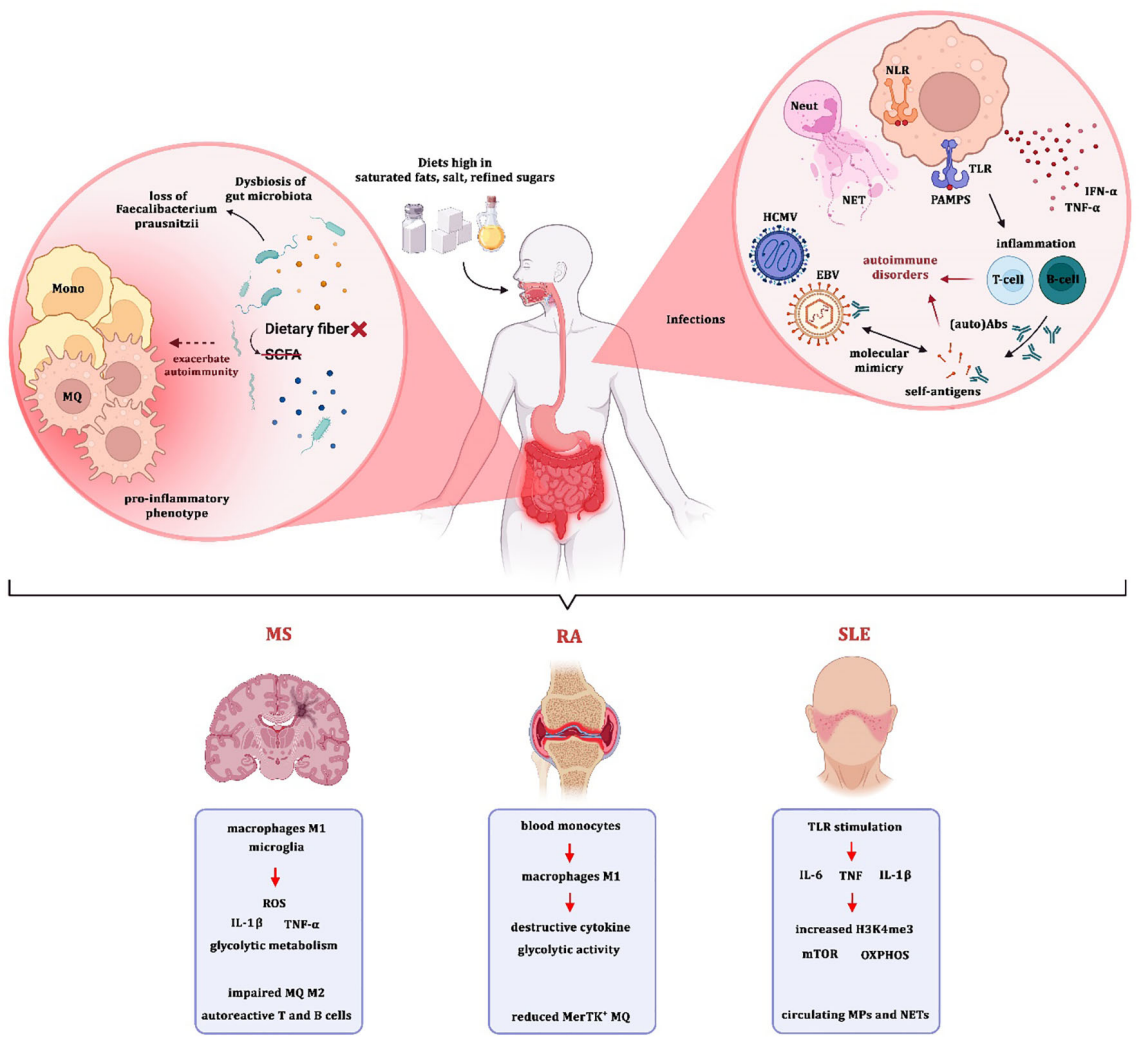
Environmental and cellular triggers of trained immunity in autoimmune diseases.

Diet constitutes another critical environmental factor. Diets high in saturated fats, salt, and refined sugars promote a pro-inflammatory state that enhances innate immune training. Conversely, anti-inflammatory diets like the Mediterranean diet, rich in fiber and polyphenols, reduce inflammation and promote immune tolerance through metabolic and epigenetic reprogramming of innate immune cells. Additionally, diet-driven alterations in the microbiota affect SCFA production, which directly modulates monocyte and macrophage differentiation and trained immunity (99) (**Figure 2**). Taken together, environmental triggers such as infections, microbiota alterations, and diet collectively shape long-term innate immune reprogramming, thereby influencing autoimmune susceptibility and disease progression.

### 4.2 Hyper-reactive monocytes/macrophages in rheumatoid arthritis, systemic lupus erythematosus, and multiple sclerosis

Monocytes and macrophages are central mediators of immune regulation and tissue inflammation in autoimmune diseases. In SLE, these cells display a hyper-inflammatory phenotype, even during remission. Upon TLR stimulation, they secrete elevated levels of IL-6, TNF, and IL-1β, linked to epigenetic changes (e.g., increased H3K4me3 at inflammatory loci) and metabolic rewiring via mTOR and oxidative phosphorylation pathways. Circulating apoptotic microparticles (MPs) and NETs further reinforce this trained immunity-like phenotype (100–102).

In RA, synovial macrophages predominantly exhibit an M1 pro-inflammatory state, releasing cytokines that drive joint and bone destruction. These cells derive from infiltrating blood monocytes and display enhanced glycolytic activity and reduced MerTK^+^ anti-inflammatory macrophage populations. Such macrophages perpetuate synovial inflammation and promote autoreactive lymphocyte activation (103, 104). Similarly, in MS, macrophages and microglia contribute to demyelination and neuronal injury through pro-inflammatory mediators such as IL-1β, TNF-α, and ROS (105). Lesions display predominance of M1-like macrophages with heightened glycolytic metabolism and impaired M2 anti-inflammatory polarization. Crosstalk between these cells and autoreactive T and B lymphocytes amplifies neuroinflammation and accelerates disease progression (106–108) (**Figure 2**).

Multiple external and internal factors contribute to the induction of trained immunity and its pathogenic role in autoimmunity. (Left) Gut microbiota and diet: Dysbiosis, characterized by loss of beneficial bacteria such as Faecalibacterium prausnitzii and reduced short-chain fatty acid (SCFA) production, promotes a pro-inflammatory phenotype in monocytes and macrophages. Diets rich in saturated fats, salt, and refined sugars further exacerbate innate immune training and chronic inflammation. (Right) Infections and molecular mimicry: Viral infections such as EBV and HCMV, along with pathogen-associated molecular patterns (PAMPs) recognized by TLRs and NLRs, activate neutrophils, NET formation, and cytokine release (e.g., IFN-α, TNF-α). These events break immune tolerance and promote activation of autoreactive T and B cells via molecular mimicry, leading to autoimmune pathology. (Bottom) Disease-specific mechanisms: In multiple sclerosis (MS), pro-inflammatory M1 macrophages and microglia produce ROS, IL-1β, TNF-α, and exhibit glycolytic metabolism, while impaired M2 polarization supports autoreactive lymphocyte activation. In rheumatoid arthritis (RA), blood monocytes differentiate into M1 macrophages with destructive cytokine release, enhanced glycolysis, and reduced MerTK^+^ anti-inflammatory macrophages. In systemic lupus erythematosus (SLE), TLR stimulation drives secretion of IL-6, TNF, and IL-1β, accompanied by epigenetic remodeling (H3K4me3), mTOR activation, and OXPHOS, with circulating microparticles (MPs) and neutrophil extracellular traps (NETs) further amplifying inflammation.

### 4.3 Role in flare-ups and chronic inflammation

Trained immunity perpetuates chronic inflammation and mediates disease flare-ups in autoimmune disorders (96, 109). Initial exposure to stimuli such as β-glucan, LPS, or fungal components induces epigenetic marks (e.g., H3K4me3, H3K27ac) and metabolic rewiring (e.g., glycolysis, mTOR/HIF-1α activation) in innate immune cells (110). This establishes a memory-like state that persists during remission, rendering monocytes and macrophages hyper-responsive to subsequent triggers, even if unrelated to the initial stimulus (111). Consequently, minor perturbations provoke exaggerated inflammatory responses, fueling tissue damage and disease relapses.

Recent studies in lupus-prone murine models demonstrate that inflammatory stimuli induce epigenetic remodeling at the level of hematopoietic stem and progenitor cells (HSPCs), leading to sustained myelopoietic bias toward inflammatory monocytes. Increased H3K4me3 enrichment at promoters of cytokine genes and enhanced mTOR signaling have been detected in circulating monocytes from patients with SLE and RA, supporting the presence of trained immunity–like signatures in human autoimmune disease (112, 113). However, while these findings strongly suggest involvement of trained immunity, mechanistic causality in human disease remains under investigation.

### 4.4 Cross-talk with adaptive immunity

Although trained immunity has traditionally been considered an innate phenomenon, accumulating evidence demonstrates that it profoundly shapes adaptive immune responses. Trained monocytes and macrophages exhibit enhanced antigen-presenting capacity through upregulation of MHC-II and co-stimulatory molecules such as CD80 and CD86, thereby promoting more robust priming of naïve T cells and skewing T-helper polarization (114, 115). These interactions highlight the functional bridge between innate memory and adaptive immunity.

Recent work has demonstrated that innate lymphoid cells, particularly ILC2s, exhibit durable epigenetic and transcriptional reprogramming following IL-33 exposure or helminth infection, acquiring a memory-like phenotype characterized by enhanced IL-5 and IL-13 production upon secondary challenge (116, 117). Chromatin accessibility analyses reveal enrichment at type 2 cytokine loci, supporting stable epigenetic imprinting.

Importantly, tumor metabolism modulates ILC plasticity. Lactate accumulation and altered lipid availability in the tumor microenvironment influence ILC2 cytokine output and may redirect type 2 immunity toward either tumor-promoting or tumoricidal programs depending on metabolic context (118, 119). These findings indicate that tumor metabolism can shape innate immune memory-like states, potentially influencing cancer progression. Emerging evidence suggests that type 2 immunity, traditionally associated with immune suppression, can under defined conditions enhance tissue remodeling and immune infiltration, contributing to antitumor effects. This underscores the non-classical and context-dependent role of type 2 immune memory in cancer biology.

The role of type 2 cytokines in cancer is increasingly recognized as context-dependent. Traditionally, IL-4 and IL-13 were considered tumor-promoting due to their association with M2 macrophage polarization and immune suppression. However, emerging evidence suggests that type 2 immunity may exert tumoricidal functions under specific metabolic and microenvironmental conditions (118). In certain models, type 2 cytokines enhance tissue repair programs that improve immune infiltration and facilitate antitumor responses (120). Therefore, type 2 immunity represents a non-classical and underappreciated arm of immunological memory that may either restrain or promote tumor development depending on the immunometabolic context.

Collectively, these findings underscore that trained immunity not only reshapes innate cell function but also dynamically influences adaptive immune polarization and tumor immunity, reinforcing its dual regulatory role in cancer and autoimmunity.

## 5 Therapeutic modulation

### 5.1 Reprogramming trained immunity for therapy

Harnessing trained immunity offers therapeutic potential in both cancer and autoimmune diseases. Its long-lasting reprogramming of innate immune cells can either amplify or suppress inflammation, depending on the clinical context (121). In cancer, enhancing trained immunity through targeted interventions can potentiate antitumor responses by reprogramming myeloid and NK cells toward pro-inflammatory and cytotoxic phenotypes, thereby improving immune surveillance and complementing adaptive immunotherapies such as immune checkpoint inhibitors (122). For example, metabolic reprogramming via modulation of glycolytic flux and mitochondrial function in monocytes and macrophages has been shown to strengthen antitumor activity while reshaping the tumor microenvironment to support effective T cell infiltration and activation (123). Conversely, in autoimmune and autoinflammatory disorders, therapeutic strategies seek to attenuate aberrant trained immunity by dampening pro-inflammatory signaling and epigenetic modifications within myeloid cells. This can be achieved by targeting upstream regulators such as mTOR or HIF-1α, thereby reducing hyper-responsiveness and restoring immune tolerance. Such approaches hold promise in rebalancing immune homeostasis while preventing the recurrence of inflammatory flares in diseases like RA, SLE, and MS (124, 125).

### 5.2 Small molecules and immunomodulators (β-glucan, statins, BCG derivatives)

It is important to distinguish between preclinical evidence, early-phase clinical trials, and established therapeutic applications when evaluating trained immunity modulators. While β-glucan-induced training is supported by robust *in vitro* and murine data, clinical oncology studies remain largely exploratory (126). In contrast, intravesical BCG in non-muscle invasive bladder cancer represents an established clinical application with decades of outcome data (127). Statins demonstrate immunomodulatory effects primarily supported by observational and mechanistic studies rather than definitive trained immunity trials (128).

To avoid redundancy, detailed mechanistic aspects of BCG- and β-glucan-induced trained immunity are described in Sections 1 and 3. Here, we focus primarily on their translational applications and levels of clinical evidence.

Several small molecules and immunomodulatory agents are being investigated to fine-tune trained immunity. β-glucan, a fungal-derived polysaccharide, is one of the most well-characterized inducers of trained immunity. It enhances innate immune responses through epigenetic remodeling (e.g., H3K4me3) and metabolic shifts toward glycolysis, improving pathogen clearance and antitumor immunity (129). Clinical studies have demonstrated its potential to augment responses to vaccines and immunotherapies in oncology settings (130–132).

Statins, widely used for lipid-lowering, exert pleiotropic effects on innate immunity by modulating cholesterol biosynthesis pathways crucial for membrane organization and immune receptor signaling (133). Beyond their cardiovascular benefits, statins reduce monocyte hyperactivation and pro-inflammatory cytokine release, suggesting their potential utility in dampening maladaptive trained immunity in autoimmune disorders (115).

**Figure 3:**
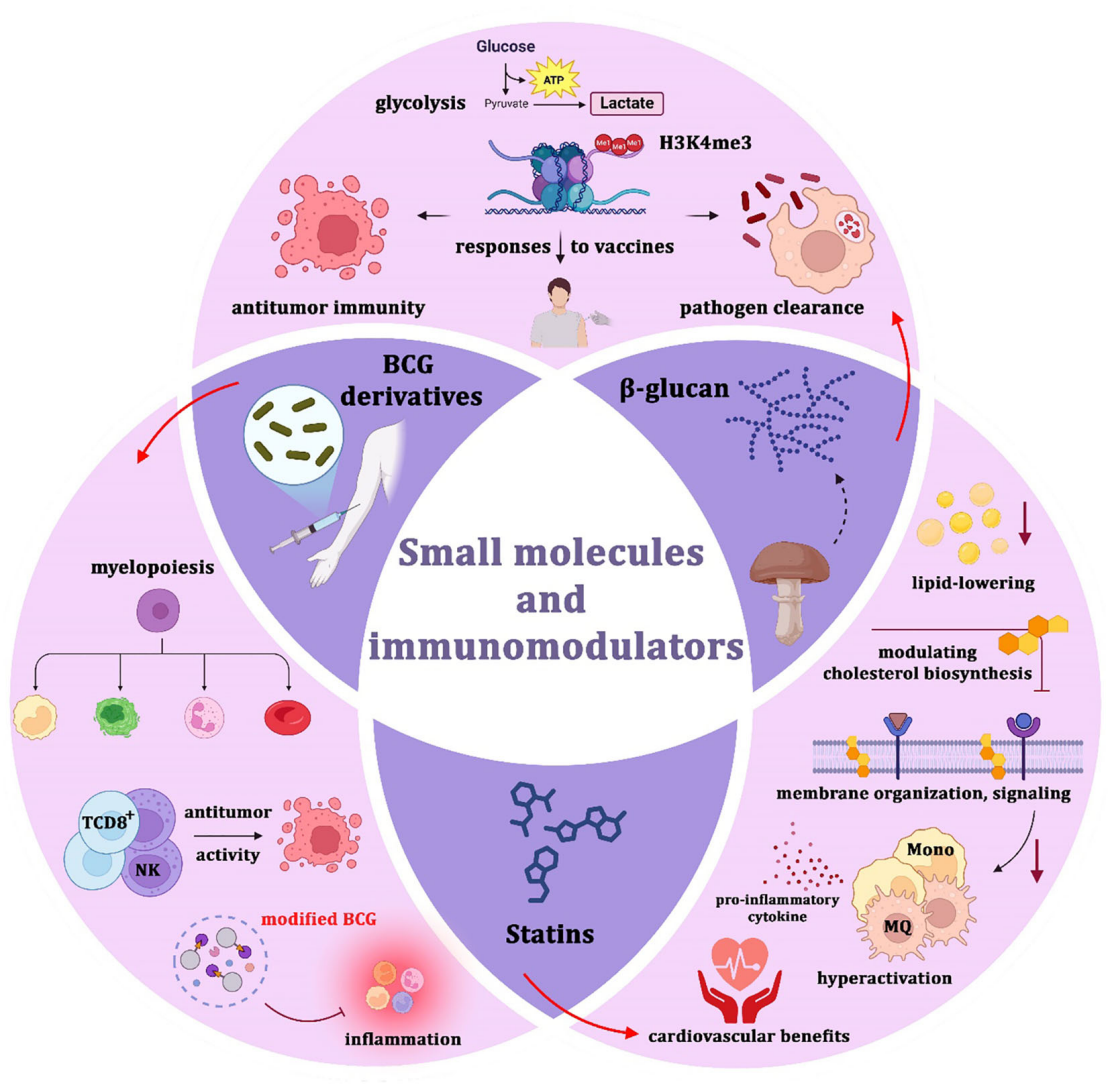
Therapeutic modulation of trained immunity using small molecules and immunomodulators. β-glucan enhances glycolysis and histone modifications (e.g., H3K4me3), thereby boosting antitumor immunity, vaccine responsiveness, and pathogen clearance. BCG derivatives stimulate myelopoiesis, augment CD8^+^ T cell and NK cell activity, and exert potent antitumor effects, although modified BCG formulations may also induce inflammation. Statins, beyond their lipid-lowering properties, influence cholesterol biosynthesis, membrane organization, and signaling, thereby modulating macrophage hyperactivation and pro-inflammatory cytokine release while providing cardiovascular protection. Collectively, these interventions highlight the therapeutic potential of pharmacological reprogramming of trained immunity, while underscoring the importance of balancing efficacy with the risk of excessive inflammation or autoimmunity.

BCG derivatives represent another therapeutic avenue, capitalizing on the capacity of Bacillus Calmette–Guérin to induce trained immunity in hematopoietic stem and progenitor cells (HSPCs) (134). Intravenous BCG has been shown to boost myelopoiesis and potentiate NK and CD8^+^ T cell responses in cancer, whereas modified BCG formulations aim to preserve beneficial immune activation while minimizing excessive inflammation (135, 136). Current research focuses on engineering BCG-based agents with enhanced specificity to tumor-associated antigens or improved safety profiles to mitigate systemic immune activation (137–139) (**Figure 3**) (**Table 1**).

**Table 1:** Therapeutic strategies targeting trained immunity in cancer and autoimmune diseases intervention.

Intervention	Primary target/pathway	Disease context	Evidence level	Model/system	Clinical status	Key references
β-glucan	Dectin-1 → Akt–mTOR–HIF-1α → glycolysis, H3K4me3	Cancer	Strong preclinical	Murine melanoma, human monocytes	Early clinical trials (adjuvant)	(55, 130–132)
β-glucan	Same pathway	Autoimmunity	Limited, context-dependent	*In vitro* monocyte models	Not established	(140)
BCG (intravesical)	PRR activation, HSPC reprogramming	NMIBC	Established clinical efficacy	Human RCTs	Approved therapy	(71–77)
Intravenous BCG	HSPC reprogramming, myelopoiesis	Solid tumors	Preclinical	Murine models	Experimental	(78, 79)
Statins	Mevalonate pathway inhibition	Autoimmune diseases	Observational + small clinical studies	RA/SLE cohorts	Adjunctive	(115, 133)
mTOR inhibitors (rapamycin)	mTORC1 inhibition	Autoimmunity	Mechanistic + clinical	Human SLE studies	Off-label	(124, 125)
TIbVs	Epigenetic + metabolic reprogramming	Infection, cancer	Early translational	Human vaccination studies	Emerging	(141–144)

### 5.3 Risks of unintended activation (e.g., triggering autoimmunity while targeting cancer)

Despite its therapeutic promise, modulation of trained immunity carries inherent risks. Amplifying innate immune responses in cancer may inadvertently trigger pathological hyperactivation in predisposed individuals, increasing the likelihood of autoimmune flares (139, 145). For instance, β-glucan–induced epigenetic reprogramming, while beneficial for antitumor immunity, may also heighten inflammatory responses in patients with underlying autoimmunity (140). Similarly, the use of BCG derivatives or other innate immune activators must be carefully monitored to avoid systemic cytokine storms or uncontrolled immune activation (134).

Moreover, dampening trained immunity in autoimmune diseases could impair host defense mechanisms against infections and malignancies. For example, excessive inhibition of mTOR or HIF-1α pathways may compromise innate immune surveillance, increasing susceptibility to opportunistic infections or reducing the efficacy of concurrent cancer therapies. These dual risks underscore the need for precision approaches that tailor therapeutic modulation of trained immunity based on individual immune profiles, disease context, and biomarker-guided monitoring (146) (**Table 1**).

Given that pathways such as mTOR and HIF-1α are shared between trained immunity induction and tumor metabolism, indiscriminate activation may risk promoting tumor growth under specific metabolic conditions (147). Therefore, therapeutic modulation requires biomarker-guided patient selection and careful longitudinal monitoring.

## 6 Future perspectives

### 6.1 Trained immunity-based vaccines

The concept of trained immunity-based vaccines (TIbVs) has emerged as a promising approach to harness the nonspecific immune-enhancing effects of innate memory to provide broad-spectrum protection against infectious diseases and cancer (141). Unlike classical vaccines that primarily rely on adaptive immune responses, TIbVs aim to reprogram innate immune cells, such as monocytes, macrophages, and NK cells, through epigenetic and metabolic remodeling, thereby establishing a heightened state of readiness for subsequent pathogen encounters (142).

BCG vaccination is a well-documented example, demonstrating not only protection against tuberculosis but also cross-protection against unrelated infections and even beneficial effects in cancer therapy. Current research focuses on designing next-generation TIbVs that use safer and more defined microbial components, such as attenuated mycobacterial derivatives, β-glucans, or synthetic PRR ligands, to induce targeted innate reprogramming with minimal inflammatory risk (143). Furthermore, TIbVs hold potential in oncology by priming the innate immune compartment to enhance tumor recognition, reshape the tumor microenvironment, and synergize with adaptive immune-based therapies, such as checkpoint blockade or CAR-T cell therapy. As such, TIbVs represent a bridge between innate and adaptive immunity, offering a versatile platform for prophylactic and therapeutic interventions across multiple disease contexts (144).

### 6.2 Personalized immunomodulation

The clinical application of trained immunity requires precision immunomodulation tailored to individual immune profiles and disease states. Variations in genetic background, epigenetic landscapes, metabolic status, microbiome composition, and prior immune exposures profoundly influence how individuals respond to trained immunity stimuli (144). In cancer, personalized strategies could involve selectively enhancing trained immunity in patients with immunosuppressive tumor microenvironments to augment immunotherapy efficacy. Conversely, in autoimmune diseases, interventions should focus on selectively dampening aberrant innate memory while preserving host defense mechanisms.

Emerging tools such as immune profiling and single-cell transcriptomics are enabling the identification of patient-specific innate immune signatures and epigenetic markers predictive of therapeutic responsiveness. These approaches may facilitate the development of tailored regimens that balance efficacy and safety, minimize the risk of autoimmunity, and optimize treatment timing (148). Moreover, integrating biomarkers such as histone modification patterns (e.g., H3K4me3, H3K27ac) or metabolic readouts (e.g., lactate levels, glycolytic flux) into clinical practice could guide dynamic monitoring of innate immune reprogramming in real time (149).

### 6.3 Integration with multi-omics and AI for patient-specific strategies

The future of trained immunity research and therapy lies in the integration of multi-omics technologies including genomics, epigenomics, transcriptomics, proteomics, metabolomics, and microbiomics to provide a holistic view of innate immune regulation. By combining these layers of data, it becomes possible to map the complex interactions between immune cells, their metabolic states, and disease environments at an unprecedented resolution (150).

Artificial intelligence (AI) and machine learning algorithms offer powerful tools for analyzing these high-dimensional datasets, enabling the identification of predictive biomarkers, novel therapeutic targets, and optimal combinations of trained immunity modulators. For instance, AI-driven models can stratify patients based on their innate immune reprogramming potential, predict their response to TIbVs or β-glucan-based adjuvants, and even simulate the outcomes of therapeutic interventions to reduce trial-and-error in clinical decision-making (151).

For example, integration of single-cell ATAC-seq with metabolomic profiling has enabled identification of persistent chromatin accessibility signatures associated with glycolytic bias in trained monocytes (152). Machine-learning algorithms trained on multi-omics datasets have been used to predict responsiveness to BCG-based interventions and to stratify patients according to innate immune reprogramming potential. Such approaches reduce reliance on empirical treatment selection and support biomarker-driven immunomodulation strategies (153).

The integration of multi-omics and AI-driven analytics will also accelerate drug discovery pipelines by uncovering context-specific regulators of trained immunity, guiding the development of small molecules or biologics that fine-tune innate immune responses. Ultimately, these technologies will facilitate personalized, patient-specific immunomodulation, allowing clinicians to strategically induce or suppress trained immunity in alignment with individual disease profiles and therapeutic goals.

## 7 Conclusion

Trained immunity represents a paradigm shift in our understanding of innate immune memory, revealing its capacity to influence both protective and pathological immune responses. By driving epigenetic and metabolic reprogramming in innate immune cells, trained immunity enhances antitumor activity and supports the efficacy of immunotherapies such as checkpoint blockade and BCG vaccination. However, its maladaptive activation can sustain chronic inflammation and exacerbate autoimmune diseases including rheumatoid arthritis, systemic lupus erythematosus, and multiple sclerosis.

Therapeutic modulation of trained immunity therefore holds significant translational potential but must be approached with precision to balance its dual roles. Interventions such as β-glucan, statins, and BCG derivatives, as well as strategies targeting mTOR and HIF-1α, exemplify the promise and complexity of manipulating innate immune memory in clinical settings. Future directions should focus on integrating multi-omics profiling, artificial intelligence, and personalized immunomodulatory approaches to develop patient-specific strategies that optimize benefits while minimizing risks.

Overall, advancing our mechanistic understanding of trained immunity and its interplay with adaptive immunity will be critical for translating these insights into innovative vaccines and therapeutic regimens capable of addressing cancer, autoimmunity, and beyond.
